# The *CCR5Δ32* (rs333) polymorphism is not a predisposing factor for severe pandemic influenza in the Brazilian admixed population

**DOI:** 10.1186/s13104-015-1299-1

**Published:** 2015-07-30

**Authors:** Alvino Maestri, Mirleide Cordeiro dos Santos, Elzemar M Ribeiro-Rodrigues, Wyller Alencar de Mello, Rita Catarina Medeiros Sousa, Sidney Emanuel dos Santos, Vinicius Albuquerque Sortica

**Affiliations:** Laboratório de Genética Humana e Médica, Universidade Federal do Pará, Cidade Universitária Prof. José da Silveira Neto, Rua Augusto Corrêa, 01, BOX: 8615, Belém, Pará CEP: 66.075-970 Brazil; Laboratory of Respiratory Viruses, Virology Section, Evandro Chagas Institute, Ananindeua, Pará Brazil; Tropical Medicine Institute, Federal University of Pará, Belém, Pará Brazil; Center of Cancer Research, Federal University of Pará, Belém, Pará Brazil

**Keywords:** A(H1N1)pdm09 infection, Influenza, *CCR5Δ32*

## Abstract

**Background:**

Recent studies have tried to identify host genetic variants that could explain severe cases and deaths in infection with Influenza A(H1N1)pdm09, especially among children and young adults. CCR5 is a chemokine receptor expressed on T cells, macrophages and dendritic cells, which is an important mediator of leukocyte chemotaxis during the immune response. A deletion mutation (Δ32) in this gene interferes with the response of immune cells, impairing viral clearance. We evaluated the *CCR5Δ32* polymorphism (rs333) in individuals of the Brazilian admixed population with a diagnosis of Influenza A(H1N1)pdm09 infection.

**Methods:**

A total of 330 subjects with a diagnosis of Influenza A(H1N1)pdm09, evaluated at health services in the northern and northeastern regions of Brazil between June 2009 and August 2010, were genotyped for the Δ32 deletion (rs333). The cases were classified according to the progression of infection into a group of hospitalized patients (n = 156) and a group of non-hospitalized patients (n = 174).

**Results:**

No significant differences in the allele or genotype frequencies of the *CCR5Δ32* polymorphism were observed between non-hospitalized and hospitalized patients (p = 0.289 and p = 0.431, respectively).

**Conclusion:**

The Δ32 deletion in the *CCR5* gene is not associated with an unfavorable outcome in patients infected with Influenza A(H1N1)pdm09 in the Brazilian admixed population.

**Electronic supplementary material:**

The online version of this article (doi:10.1186/s13104-015-1299-1) contains supplementary material, which is available to authorized users.

## Background

On April 21, 2009 [1], the Centers for Disease Control and Prevention (CDC) announced two flu cases in children from California, USA, caused by a new influenza strain originated by the quadruple reassortment between other already circulating influenza viruses [2]. The new viral subtype spread around the world, a fact that culminated in the announcement by the World Health Organization (WHO) on June 11, 2009, of the first flu pandemic in the 21st century [3]. The lethality of the new viral strain did not add to the flu-related death statistics; however, the large number of severe cases and deaths among children and young adults called the attention of the scientific community [4].

Recent studies have tried to identify host genetic variants that could explain severe cases of the disease [5]. On the basis of the cycle of viral replication of the pandemic strain in human cells, genetic variants that could influence viral clearance [6] have been identified. One genetic variant is a 32-bp deletion in the *CCR5* gene (Δ32) [7].

CCR5 is a chemokine receptor expressed on T cells, macrophages and dendritic cells, which is an important mediator of leukocyte chemotaxis during the response to chemokines. The interaction of this receptor with its ligands results in the homing of different immune cells to the sites of viral infection on the mucosal surface. Studies have shown that the Δ32 deletion in the *CCR5* gene interferes with the response of immune cells through CCL3, CCL4 and CCL5, impairing viral clearance [8–10]. In a population of 20 Canadian patients who developed severe forms of infection with the pandemic flu virus, the mutation at position 32 of the CCR5 gene (rs333) was detected in five patients and was associated with an unfavorable clinical evolution [7]. However, the same mutation evaluated in 29 Italian patients also infected with the pandemic strain was not associated with poor clinical outcome [11]. In the present study, the *CCR5Δ32* polymorphism (rs333) was investigated in individuals from a Brazilian admixed population with a diagnosis of Influenza A(H1N1)pdm09 infection.

## Methods

### Population

Between June 2009 and August 2010, the Virology Section of the Evandro Chagas Institute (Seção de Virologia do Instituo Evandro Chagas—SEVIR/IEC) received 5,427 nasal swab or nasopharyngeal aspirate samples from subjects with a clinical suspicion of flu-like illness who sought health services in the states of the northern and northeastern regions of Brazil. Of these, 1,524 samples were positive for the pandemic strain and 330 samples with a diagnosis of Influenza A(H1N1)pdm09 were randomly included in the study. All patients enrolled in the study provided their written informed consent. Underage participants (younger than 18 years n = 115) had the informed consents signed by parents to participate in the study. The study was approved by the Ethics Committee of the Center of Tropical Medicine, Federal University of Pará (Núcleo de Medicina Tropical, Universidade Federal do Pará).

### Laboratory diagnosis

Diagnostic confirmation was performed at the Laboratory of Respiratory Viruses, SEVIR/IEC, Ananindeua, Pará, using the SuperScript III™ One-Step qRT-PCR System with Platinum Taq^®^ (Invitrogen Life Technologies, Carlsbad, CA, USA) according to the protocol recommended by the CDC [12].

### Genotyping of *CCR5Δ32* (rs333)

Genomic DNA was extracted from the leukocyte aggregate found in the nasal aspirate or nasopharyngeal swab using the QIAamp DNA Mini Kit (Qiagen, Valencia, CA, USA) according to manufacturer instructions. All DNA samples were genotyped by PCR. The primers (forward: CTCCCAGGAATCATCTTTACCA and reverse: TTTTTAGGATTCCCGAGTAGCA) were designed using the Primer3 software (http://www.genome.wi.mit.edu/cgi-bin/primer/primer3) and tested with the AutoDimer software.

PCR was carried out in a final volume of 12.5 µL containing PCR buffer 1 with 3 mM of MgCl_2_, 125 mM of each dNTP, 2 U Platinum AmpliTaq DNA polymerase (Invitrogen Life Technologies, Carlsbad, CA, USA) and 10–20 ng genomic DNA. The PCR conditions were: 11 min at 95°C; 10 cycles of 1 min at 94°C, 1 min at 60°C and 2 min at 70°C; 17 cycles of 1 min at 90°C, 1 min at 60°C and 2 min at 70°C, and a final extension of 60 min at 60°C. For capillary electrophoresis, 1 mL of the PCR product was added to 8.5 mL Hi-Di formamide (Applied Biosystems, Foster City, CA, USA) and 0.5 mL GeneScan 500 LIZ Size Standard (Applied Biosystems, Foster City, CA, USA). The amplicons were separated in an ABI Prism 3130 Genetic Analyzer (Applied Biosystems, Foster City, CA, USA) and analyzed using the v3.2 GeneMapper ID software (Applied Biosystems, Foster City, CA, USA).

### Population substructure

The proportions of African, European and Amerindian genetic ancestry of the infected patients were estimated using a panel of 48 ancestry-informative markers as described previously [13].

### Statistical analysis

Allele and genotype frequencies were estimated by direct counting. Deviation from Hardy–Weinberg equilibrium was verified by Chi squared analysis. Differences in quantitative and qualitative characteristics between the groups of hospitalized and non-hospitalized patients were evaluated using the Student *t* test, Chi squared test and Fisher’s exact test. Differences in the proportions of genetic ancestry between groups were determined using the Wilcoxon–Mann–Whitney test. Fisher’s exact test was used to analyze differences in the allele and genotype frequencies of the deletion studied between the groups of patients. Logistic regression was used to assess the effect of the *CCR5* polymorphism on the severity of Influenza A(H1N1)pdm09 infection. European and African genetic ancestry and comorbidities (dichotomous variable, presence or not of comorbidity) were included in the model as confounders, as they presented differences between patients groups. Statistical analysis was performed using the SPSS 18.0 software, adopting a level of significance of p < 0.05.

## Results

The 330 patients included in the study exhibited the classical clinical symptoms of the disease and were divided according to the progression of infection into two groups: patients that presented severe acute respiratory syndrome (SARS) and were hospitalized (n = 156) following Brazilian Ministry of Health protocol [14] and patients with mild symptoms non-hospitalized (n = 174) (Table 1). There was a predominance of women among the patients studied (61.5%) and the mean patient age was 24.7 years (1–80 years). Forty-seven of the women were pregnant and 28 were hospitalized. Radiologic alterations were found in 51.9% of hospitalized patients. Most hospitalized patients had no comorbidities (59%). However, patients with comorbidities (metabolic disorders, immunosuppression and obesity) were significantly more frequent in the group of hospitalized patients. Deaths occurred in 55.7% of hospitalized patients. The population studied exhibited a mean European genetic contribution of 57.4%, a mean Native American contribution of 26.4% and a mean African contribution of 16.2% (Table 1) in agreement with previously data from Brazilian populations [13, 15]. In non-hospitalized patients the mean European contribution was 60% ranging from 4.6 to 91.9%, the mean Native American contribution was 25% ranging from 3.2 to 93.2%, and the mean African contribution was 15% ranging 2.2–61%. In hospitalized patients, genetic contribution was European 54.4% ranging from 5.9 to 93%, Native American 28% ranging from 3.5 to 90.6%, and African 17,6% ranging 2.4–67.6% (Additional files 1, 2). European and African genetic ancestry showed statistical differences between groups (p = 0.006 and p = 0.010, respectively).

**Table 1:** Epidemiological and clinical characteristics of hospitalized and non-hospitalized patients infected with Influenza A(H1N1)pdm09

Characteristics	All patients	Non-hospitalized	Hospitalized	p value
N	330	174	156	
Female sex	203 (61.5)	102 (58.6)	101 (64.7)	0.254
Age (years)	24.7 ± 15.3	23.8 ± 14.2	25.8 ± 16.5	0.270
Pregnant	47 (31.8)	19 (26.0)	28 (37.3)	0.140
Smoking	18 (5.5)	6 (3.4)	12 (7.7)	0.090
Signs and symptoms				
Fever	320 (97.0)	167 (96.0)	153 (98.1)	0.267
Cough	311 (94.2)	161 (92.5)	150 (96.2)	0.158
Shortness of breath	245 (74.2)	101 (58.0)	144 (92.3)	<0.001
Muscle aches	212 (64.2)	117 (67.2)	95 (60.9)	0.230
Rhinorrhea	197 (59.7)	113 (64.9)	84 (53.8)	0.040
Sore throat	192 (58.2)	107 (61.5)	85 (54.5)	0.198
Chills	138 (41.8)	72 (41.4)	66 (42.3)	0.864
Joint pain	118 (35.8)	64 (36.8)	54 (34.6)	0.682
Headache	63 (19.1)	40 (23)	23 (14.7)	0.057
Diarrhea	55 (16.7)	24 (13.8)	31 (19.9)	0.139
Conjunctivitis	21 (6.4)	15 (8.6)	6 (3.8)	0.076
Abnormal chest radiograph	81 (24.5)	0 (0)	81 (51.9)	<0.001
Without comorbidities	220 (66.7)	128 (73.6)	92 (59.0)	0.005
With comorbidities				
Chronic lung disorder	65 (19.6)	29 (16.7)	36 (23.1)	0.144
Chronic cardiovascular condition	22 (6.6)	10 (5.7)	12 (7.7)	0.470
Metabolic disorder	12 (3.6)	1 (0.6)	11 (7.1)	0.002
Immunosuppression	7 (2.1)	0 (0)	7 (4.5)	0.005
Obesity	8 (2.4)	1 (0.6)	7 (4.5)	0.021
Hemoglobinopathy	3 (0.9)	1 (0.6)	2 (1.3)	0.490
Chronic kidney disease	4 (1.2)	1 (0.6)	3 (1.9)	0.260
Death	87 (26.3)	0 (0)	87 (55.7)	<0.001
Genetic ancestry				
Native American	0.264 ± 0.18	0.250 ± 0.19	0.280 ± 19	0.147
European	0.574 ± 0.20	0.600 ± 0.20	0.544 ± 19	0.006
African	0.162 ± 0.11	0.150 ± 0.10	0.176 ± 11	0.010

Age and genetic ancestry were reported as the mean ± SD. All other variables are reported as number (%).

There were no significant differences in the allele or genotype frequencies of the *CCR5Δ32* polymorphism between non-hospitalized and hospitalized patients (p = 0.289 and 0.431, respectively) (Table 2). A logistic regression analysis was performed to assess the effect of *CCR5Δ32* polymorphism on infection severity controlling for European and African ancestry and the presence of comorbidities to avoid confounding effects. No association between patients carrying the Δ32 allele and severity was found (Table 3).

**Table 2:** Genotype and allele distribution of CCR5Δ32 in non-hospitalized and hospitalized patients infected with Influenza A(H1N1)pdm09

	Non-hospitalized	Hospitalized	p value
Genotype			
Wt/Wt	160 (92.0)	148 (94.9)	0.431
Wt/Δ32	13 (7.5)	8 (5.1)	
Δ32/Δ32	1 (0.6)	0 (0)	
Allele			
Wt	333 (0.96)	304 (0.97)	0.289
Δ32	15 (0.04)	8 (0.03)

**Table 3:** *CCR5Δ32* effect on A(H1N1)pdm09 infection severity

Genotypes	B	OR	CI 95%	P value
Wt/Wt^a^				
Δ32 carriers^b^	−0.582	0.559	0.22; 1.26	0.219

Covariates in Regression model: European and African ancestry and comorbidities.

^a^Reference genotype.

^b^Wt/Wt + Wt/Δ32.

## Discussion

Since the demonstration of the protective role of a 32-bp deletion in the *CCR5* gene in two individuals exposed to, but not infected with HIV [16], studies on the protective or regulatory role of this deletion have multiplied [17]. The protective role of this polymorphism in HIV infection was extensively studied and utilized as model for the development of new therapeutics for AIDS [18].

However, the presence of the deletion can be a determinant factor of morbidity and mortality in the case of other infectious diseases. In a meta-analysis of four cohorts evaluating the deletion in patients with West Nile fever, genetic deficiency of the *CCR5* gene was a strong risk factor of symptomatic arbovirus infection [19]. In another study comparing 129 patients with a diagnosis of tickborne encephalitis virus infection and 79 subjects with aseptic meningitis negative for tickborne encephalitis and 134 healthy controls, a higher frequency of the homozygous Δ32/Δ32 genotype was observed among patients with tickborne encephalitis, particularly among severe cases of the disease [20].

In the present study, no difference in the Δ32 deletion of the *CCR5* gene was observed between the groups of individuals infected with Influenza A(H1N1)pdm09 in a Brazilian admixed population, who presented the classical clinical symptoms [21]. Thus, no correlation could be established between the presence of the mutation and a more severe outcome of the disease. The present results agree with those reported in a recent study involving an Italian population [11], but disagree with the findings obtained for a Canadian population [7].

The Brazilian population was formed by extensive admixture of Native American, European and African populations, nevertheless European genetic ancestry is predominantly in Brazil [13, 15]. In the population studied the mean European contribution was around 57% and the *CCR5Δ32* allele frequency was 3.6%, similar to previously described for Brazilian populations [22]. Despite ethnic differences with the populations previously analyzed, in the present study thirteen heterozygous and one homozygous for the Δ32 deletion were found in patients with mild symptoms and eight heterozygous for this deletion were found in hospitalized group showing no evidence of this allele effect on severity.

Previous researches adopted different criteria for severe patients [7, 11], which could contribute for conflicting results among studies, but despite those differences, we emphasize the large size of the sample studied here compared to previously published studies, supporting the hypothesis that the Δ32 mutation is not a predisposing factor for severe Influenza A(H1N1)pdm09 infection.

## Conclusion

Although studies have reported an association between mutation Δ32 in the CCR5 gene and a more severe evolution of some infectious diseases, our findings demonstrate that the same cannot be confirmed for infection with Influenza A(H1N1)pdm09 in the Brazilian admixed population.

## Additional files

Additional file 1: Genetic ancestry admixture of patients infected with Influenza A(H1N1)pdm09 sorted by African ancestry. Each individual ancestry is depicted as a column, whereas color represents the proportion of ancestry estimated for that individual (African = blue; European = brown; Native American = green). (A) Non-hospitalized patients and (B) Hospitalized patients. Additional file 2: Genetic ancestry admixture of patients infected with Influenza A(H1N1)pdm09 sorted by European ancestry. Each individual ancestry is depicted as a column, whereas color represents the proportion of ancestry estimated for that individual (African = blue; European = brown; Native American = green). (A) Non-hospitalized patients and (B) Hospitalized patients.

## Abbreviations

CDC: Centers for Disease Control and Prevention
IEC: Evandro Chagas Institute
SEVIR: Virology Section
WHO: World Health Organization
