# Supplementary material for: The CCR5Δ32 (rs333) polymorphism is not a predisposing factor for severe pandemic influenza in the Brazilian admixed population
Source: BMC Res Notes. 2015 Jul 30;8:326. doi: 10.1186/s13104-015-1299-1 (PMC4520097; doi:10.1186/s13104-015-1299-1)
Supplement: Additional file 1: — Genetic ancestry admixture of patients infected with Influenza A(H1N1)pdm09 sorted by African ancestry. Each individual ancestry is depicted as a column, whereas color represents the proportion of ancestry estimated for that individual (African = blue; European = brown; Native American = green). (A) Non-hospitalized patients and (B) Hospitalized patients. [file 13104_2015_1299_MOESM1_ESM.pdf]

A

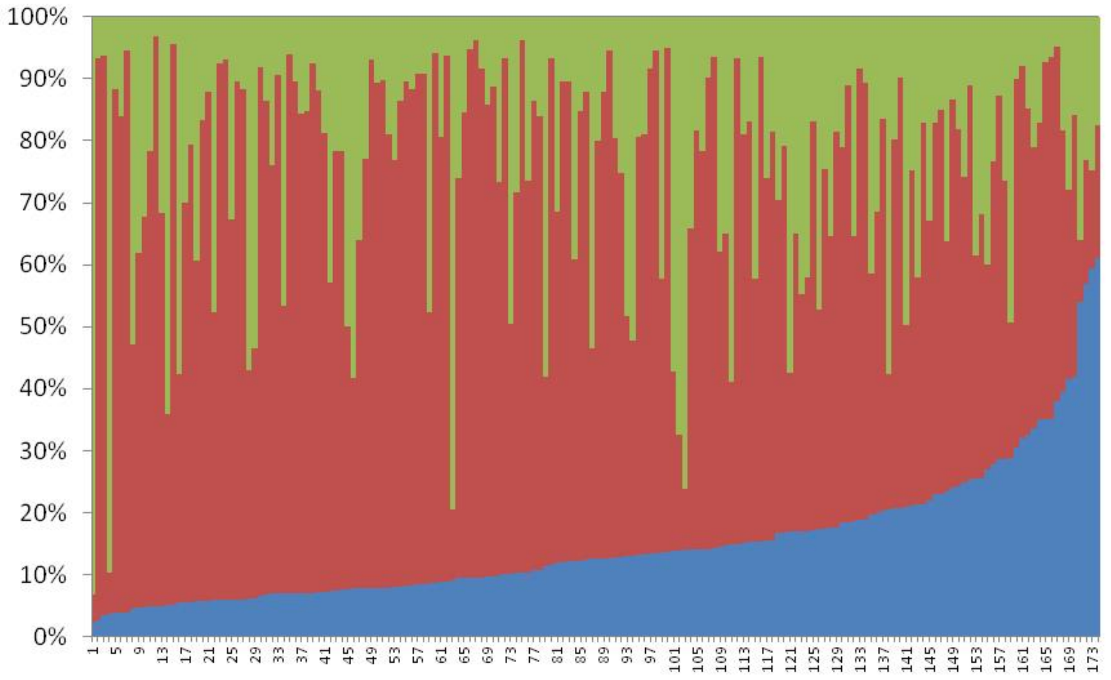

B

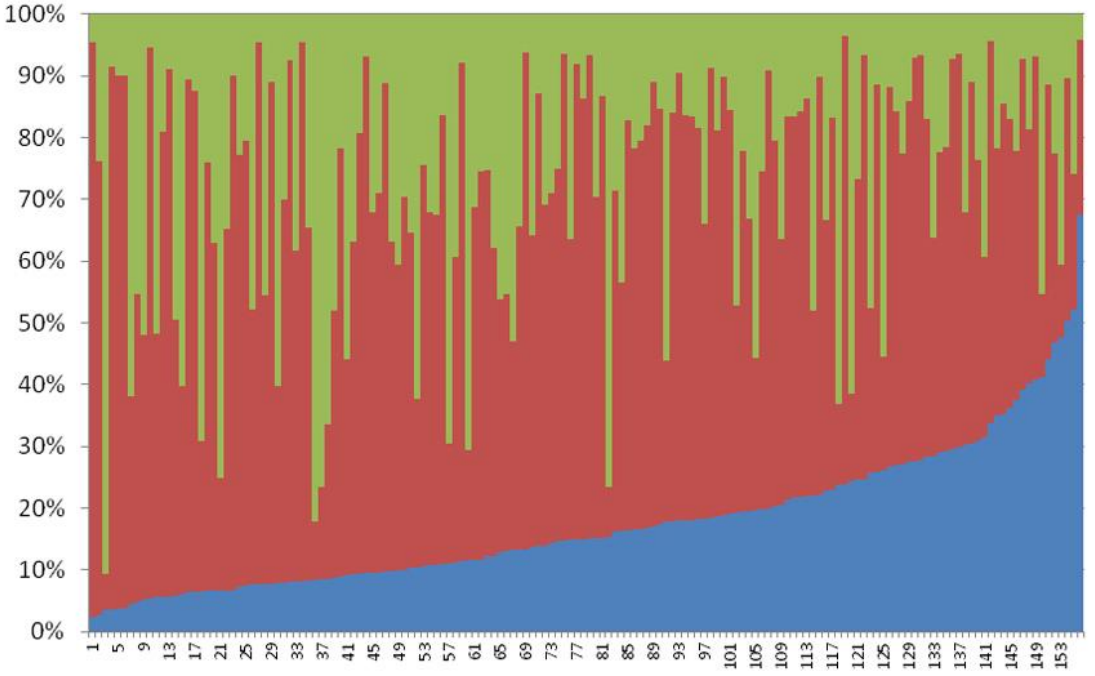

Additional File 1. Genetic ancestry admixture of patients infected with Influenza A(H1N1)pdm09 sorted by African ancestry. Each individual ancestry is depicted as a column, whereas color represents the proportion of ancestry estimated for that individual (African = blue; European = brown; Native American = green). (A) Non-hospitalized patients and (B) Hospitalized patients.
